# Is There a Reliable, Rapid, and Economic Diagnostic Approach for SLCO2A1-Related Chronic Enteropathy?

**DOI:** 10.3390/jcm15062433

**Published:** 2026-03-22

**Authors:** Rongbei Liu, Yujuan Fu, Jie Mao, Zhinong Jiang, Qian Cao, Lingna Ye

**Affiliations:** 1 Department of Gastroenterology, Sir Run Run Shaw Hospital, School of Medicine, Zhejiang University, Hangzhou 310027, China; liurongbei@zju.edu.cn; 2 Center of Inflammatory Bowel Disease, Sir Run Run Shaw Hospital, School of Medicine, Zhejiang University, Hangzhou 310027, China; 3315013@zju.edu.cn (Y.F.); 3411014@zju.edu.cn (J.M.); 3200039@zju.edu.cn (Z.J.); 3 Department of Pathology, Sir Run Run Shaw Hospital, School of Medicine, Zhejiang University, Hangzhou 310027, China

**Keywords:** SLCO2A1, CEAS, immunohistochemical staining

## Abstract

**Background**: Chronic enteropathy associated with SLCO2A1 (CEAS) is a rare genetic disorder that is prone to misdiagnosis and characterized by significant challenges in achieving an early diagnosis. Current diagnosis relies on clinical manifestation combined with genetic sequencing. This study aimed to evaluate immunohistochemical (IHC) staining for SLCO2A1 protein deficiency as a diagnostic alternative, in addition to clinical pathological features. **Method**: Ten patients diagnosed with CEAS between January 2018 and August 2024 were enrolled. Clinicodemographic data, endoscopic findings, and treatment history were collected. Whole-exome sequencing identified SLCO2A1 variants. IHC staining for SLCO2A1 protein was performed from small intestine lesions and accessible GI sites. **Results**: Complete absence of SLCO2A1 protein expression was demonstrated by IHC in 9/10 patients, with significantly reduced expression in 1/10. This protein deficiency was consistently observed not only in the small intestine but also in the gastric antrum, duodenum, and terminal ileum. Genetic analysis revealed 7 novel SLCO2A1 variants among a total of 11 variants. A median diagnostic delay of 15 years (IQR 6–24) was observed. Ileal involvement and hypoalbuminemia (median albumin 28.02 g/L, IQR 22.1–33.0) were present in all patients. Common symptoms included abdominal pain (70%), melena (60%), and ileus (50%). **Conclusions**: The diagnosis of CEAS has been time-consuming and challenging. Detection of SLCO2A1 protein deficiency via IHC in either the disease-predominant small bowel or accessible non-lesional upper/lower GI mucosa demonstrates high diagnostic sensitivity for CEAS. This method could provide a practical, cost-effective alternative to genetic sequencing, particularly in resource-limited settings, and has the potential to significantly reduce diagnostic delays for this condition.

## 1. Introduction

Chronic enteropathy associated with SLCO2A1 (CEAS) is a rare monogenic disorder characterized by gastrointestinal symptoms such as abdominal pain, anemia, and gastrointestinal bleeding [1,2]. It was initially considered a type of nonspecific multiple ulcers of the small intestine before SLCO2A1 gene mutations were identified [2]. The SLCO2A1 gene encodes the organic anion-transporting polypeptide 2A1 (OATP2A1), which is a prostaglandin transporter that is crucial for regulating prostaglandin E2 (PGE2) levels in the gastrointestinal tract. These mutations lead to impaired prostaglandin transport and inactivation, resulting in chronic mucosal inflammation and damage [3,4,5].

Diagnosing CEAS is challenging due to its rarity and the overlap of symptoms with other gastrointestinal conditions, such as Crohn’s disease (CD) and cryptogenic multifocal ulcerous stenosing enteritis (CMUSE) and other small bowel ulcerative disorders [6]. The clinical understanding of CEAS primarily relies on individual case reports, which has led to inadequate disease recognition. Consequently, the median diagnostic delay exceeds 10 years [7,8]. Frequent misdiagnosis often results in inappropriate treatments, disease progression, and a diminished quality of life [2,9,10]. Therefore, the early identification and diagnosis of CEAS are crucial for effective management, prevention of medical resource waste, and improvement of patient outcomes.

The diagnosis of CEAS has relied on clinical manifestation combined with genomic DNA sequencing to identify SLCO2A1 mutations. Although accurate, this genetic method is costly, time-consuming, and frequently inaccessible in resource-limited healthcare settings [11]. As a result, there is a need for alternative methods that can still accurately identify patients with CEAS. By directly assessing protein expression, immunohistochemical (IHC) staining of SLCO2A1 protein in these biopsy samples may provide a more practical, rapid, and cost-effective diagnostic alternative.

Small intestinal ulcers are the main lesions in CEAS [1], and obtaining biopsy samples from these areas often requires invasive procedures such as enteroscopy. These methods can be difficult to perform, particularly in hospitals below the Grade A tertiary level, and with risks such as internal bleeding, bowel perforation and complications from anesthesia [12]. To overcome these diagnostic challenges, as CEAS is caused by germline mutations in the SLCO2A1 gene, we postulated that the resulting protein expression defect should be systemic and constitutive, affecting SLCO2A1-expressing cells throughout the gastrointestinal tract, not solely at the sites of active ulceration. Therefore, we aimed to evaluate whether this deficiency could be detected in the more readily accessible epithelium of the gastric antrum, duodenum, and terminal ileum obtained during routine endoscopy, even if these areas appeared endoscopically normal.

Therefore, this study aims to evaluate the utility of IHC staining as a diagnostic tool for CEAS, to assess the impact of tissue sampling location within the gastrointestinal tract on the results, and to summarize the clinical features observed in patients diagnosed with CEAS at our hospital. We hope to provide a viable diagnostic alternative that can be implemented in routine clinical practice, particularly in resource-limited settings where genetic testing is not readily available. Additionally, this approach may facilitate earlier diagnosis and treatment, potentially improving patient outcomes.

## 2. Materials and Methods

### 2.1. Study Participants and Clinical Data

Ten patients diagnosed with CEAS from January 2018 to August 2024 at Sir Run Run Shaw Hospital were enrolled. The CEAS diagnosis was based on acknowledged clinical criteria and genetic identification of SLCO2A1 gene variants. Clinicodemographic characteristics were collected from medical records, including gender, age at symptom onset, age at CEAS diagnosis, family history, parental consanguineous marriage, history of surgery, symptoms, endoscopic results, endoscopic and surgical pathology, laboratory test results, CT or MRE results, treatment history and effect, and past diagnosis. Informed consent was obtained from all subjects. Primary hypertrophic osteoarthropathy (PHO) diagnosis was based on typical clinical manifestations including digital clubbing, pachydermia, and periostosis by X-ray [13]. We also captured data on PHO manifestations. The study was approved by the Clinical Research Ethics Committee of Sir Run Run Shaw Hospital Affiliated to Zhejiang University School of Medicine (number: 2024Y1107).

### 2.2. Genomic DNA Preparation and Whole-Exome Sequencing

Peripheral blood samples were collected using EDTA-coated tubes and sent to Kingmed Co., Ltd. (Hangzhou, China) for genomic DNA extraction and whole-exome sequencing. Genomic DNA was extracted from peripheral blood using the QIAsymphony DSP DNA Mini Kit (Qiagen, Germany) according to the manufacturer’s instructions. Whole-exome sequencing was performed on an Illumina NovaSeq 6000 sequencing platform, and the sequencing data were processed for base calling and alignment to the human reference genome hg19 to identify and further confirm the sequence and mutations of SLCO2A1 of each subject.

### 2.3. Immunohistochemical Staining

First, 4 μm thick sections of the paraffin-embedded tissues were cut and spread with distilled water on slides. Then, the slides were dried overnight at 50 °C, followed by deparaffinization and hydration through xylene and graded ethanol to distilled water, with endogenous peroxidase blocked in 0.3% H_2_O_2_ in 95% ethanol for 5 min during hydration. Antigen retrieval was performed using heat-induced epitope retrieval (HIER) in citrate buffer (pH 6.1) with a pressure boiler at 110 °C for 20 min, and then cooled to 90 °C. For IHC staining at room temperature, sections were incubated with Ultra V Block for 5 min, then with the primary antibody (SIGMA, St. Louis, MO, USA, HPA013742) for 30 min, followed by incubation with a labeled polymer for 30 min, and development with 3,3′-diaminobenzidine (DAB) for 5 min. Finally, the sections were counterstained with hematoxylin for 5 min, blued in lithium carbonate solution, dehydrated through graded ethanol and xylene, and mounted with Pertex. The sections were observed and the extent of SLCO2A1 expression was evaluated under a microscope. Positive staining was recognized as a brown color in the nucleus of cells. A known SLCO2A1-positive case was used as positive control.

### 2.4. Statistical Analysis

Non-normally distributed data are presented as median with interquartile range (IQR). Categorical variables are presented as frequency (n) and percentage (%). SPSS 22.0 (IBM, Armonk, NY, USA) was used for data analysis.

## 3. Results

### 3.1. Clinical Characteristics of CEAS Patients

The clinical characteristics of 10 CEAS patients are summarized in Table 1. The median age at symptom onset was 25 years (IQR 17–36), while the median age at diagnosis was 41 years (IQR 39–44), indicating a substantial diagnostic delay with a median interval of 15 years (IQR 6–24) from symptom onset to diagnosis. None of the patients had a family history of CEAS, but 40% had parental consanguineous marriages. Half of the patients had undergone bowel resection, with four patients requiring one surgery and one patient undergoing two surgeries. The median time from symptom onset to the first bowel resection surgery was 13 years (IQR 1–26).

The common symptoms reported were abdominal pain (70%), melena (60%), ileus (50%), and hypoalbuminemia (100%). Involvement of the ileum was observed in all patients, while other parts of the gastrointestinal tract, such as the duodenum, jejunum, and colon, were less frequently involved. None of the female patients and two of the male patients were diagnosed with primary hypertrophic osteoarthropathy (PHO), which included clinical manifestations such as digital clubbing, pachydermia, periostosis, and joint pain, all present in 100% of the PHO patients.

Laboratory data revealed hypoalbuminemia in all patients, with a median albumin level of 28.02 g/L (IQR 22.1–33.0). Hemoglobin levels were also reduced, with a median of 70 g/L (IQR 39.8–100.5), and inflammatory markers, such as C-reactive protein (CRP) and erythrocyte sedimentation rate (ESR), were mildly elevated in patients.

Notably, 90% of the patients had initially been misdiagnosed with Crohn’s disease, and 40% were misdiagnosed with CMUSE. Therefore, the treatment history included mesalazine (40%), methylprednisolone (70%), azathioprine (20%), thalidomide (40%), biologics (20%), and enteral nutrition (60%). We found that aside from the partial symptom relief achieved with enteral nutrition, other medications were ineffective in managing the symptoms.

### 3.2. Genetic Analysis

SLCO2A1 gene mutations identified in the 10 CEAS patients were summarized in Table 2. Several types of mutation were observed with corresponding amino acid changes, including splicing site (SS), missense (MIS), nonsense (NON), and frameshift (FS) mutations. The most common mutation identified was the splicing site mutation c.940 + 1G > A, observed in both homozygous and compound heterozygous forms. In total, seven patients had homozygous mutations, while three had compound heterozygous mutations.

In comparison with existing case reports of CEAS, 7 of the 11 variants detected from these 10 patients, whether homozygous or heterozygous, had not been reported previously in CEAS patients. Interestingly, 4 of the 11 newly discovered variants, c.1633A(2 > 1) (one mutant allele in patient 3), c.581A > G and c.268_279del (both in patient 4), and c.910_914del (one mutant allele in patient 5), had not been previously reported, even in the Human Gene Mutation Database (HGMD).

We found that only two patients exhibited the same mutation type, indicating a high diversity of SLCO2A1 variants in CEAS patients. All of these mutations impact the amino acid sequence, which may affect the final protein structure and protein expression. Consequently, we performed immunohistochemical staining on these patients to further investigate the potential functional consequences.

### 3.3. Immunohistochemical Staining in Different Sites

**Figure 1:**
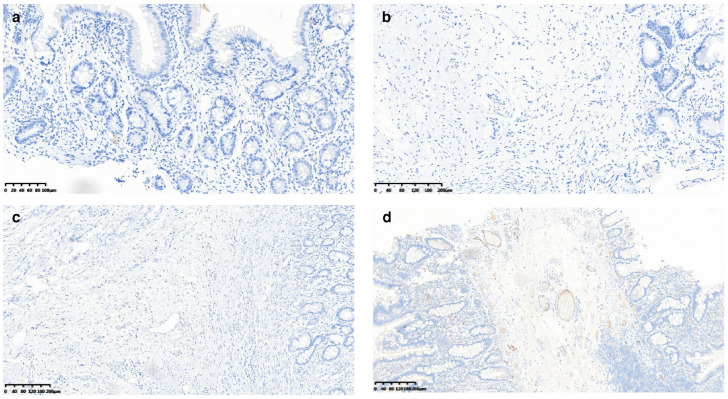
Immunohistochemical staining in different sites. (**a**) non-lesional terminal ileum, (**b**) gastric antrum, (**c**) ileum, (**d**) positive control.

To investigate the impact of gene mutations on protein expression, we performed immunohistochemical (IHC) staining of the SLCO2A1 protein on small intestine biopsy samples and surgical specimens from CEAS patients. The results showed that SLCO2A1 protein expression was entirely absent in nine patients, while it was significantly reduced in one patient (Figure 1).

**Figure 2:**
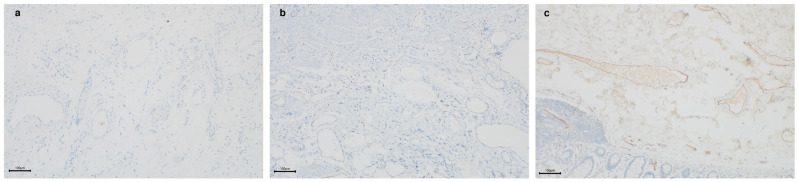
Different SLCO2A1 expression results. (**a**) SLCO2A1 protein expression was entirely absent, (**b**) SLCO2A1 protein expression was significantly reduced, (**c**) positive control. The complete absence of specific nuclear brown staining was scored as “absent,” a pronounced qualitative reduction compared to the strong homogeneous staining in positive controls was scored as “reduced,” and clear nuclear staining in the majority of cells was considered “positive”.

Due to the difficulty of obtaining samples from the small intestine and the fact that many patients initially undergo gastroscopy and colonoscopy, this raises the question of whether protein deficiency might be observed in other gastrointestinal regions accessible by gastroscopy and colonoscopy. To address this, we conducted IHC staining on tissue samples from the gastric antrum, duodenum, and terminal ileum obtained during gastroscopy or colonoscopy. The results indicated that SLCO2A1 expression in these regions was consistent with the findings in the small intestine (Figure 2). The symptoms and IHC staining results of each CEAS patient are listed in Table 3.

## 4. Discussion

The findings of this study highlight the significant diagnostic value of immunohistochemical (IHC) staining of the SLCO2A1 protein in patients with CEAS. The diagnosis of CEAS is challenging due to its clinical symptoms being similar to those of CD, CMUSE and other small bowel ulcerative disorders [6]. Additionally, as a rare disease, conventional methods such as endoscopy, imaging, and pathological examination often fail to provide a definitive diagnosis for this rare disease, leading to frequent delays. To address this unmet need, we conducted this study to evaluate the diagnostic value of immunohistochemical (IHC) staining for the SLCO2A1 protein in gastrointestinal biopsy specimens.

The diagnosis of CEAS remains challenging. Consistent with previous studies [7,8], the patients in this study exhibited a prolonged interval from the onset of symptoms to the confirmed diagnosis, with an average delay of 15 years, revealing the severe delay in diagnosis. Multiple factors might contribute to this diagnostic delay, for example, late definition of CEAS and the unavailability of convenient diagnostic assays. As awareness of CEAS increases among clinicians, genetic testing, particularly whole-exome sequencing, is considered the gold standard for diagnosing CEAS [2]. However, due to factors such as cost, long turnaround time, and low positivity rate, it is not routinely used as a diagnostic tool for distinguishing small bowel ulcers. To differentiate CEAS from other conditions presenting as small bowel ulcers, researchers have begun exploring alternative, more accessible diagnostic methods. Matsuno investigated the feasibility of using prostaglandin E major urinary metabolites (PGE-MUMs) as biomarkers to differentiate CEAS from CD, and proposed an optimal PGE-MUM cut-off value for this distinction [14]. However, this study focused on patients with remission CD, and the proposed cut-off value cannot be generalized to other diseases, including active CD. Some researchers have compared the immunohistochemical expression of SLCO2A1 protein in different diseases, although the sample size for CEAS remains small [15]. In this study, we found that the results of immunohistochemistry were consistent with genetic findings, as IHC staining detected the loss of SLCO2A1 protein expression in almost all cases.

Considering the difficulty of obtaining biopsies from the small intestine, we further compared the IHC results from different sites, a comparison not previously addressed in the literature. An important finding in our study was the consistency of IHC results obtained from biopsy specimens taken from different parts of the gastrointestinal tract, including the ileum, duodenum, and small intestine. This suggests that IHC for SLCO2A1 protein expression is not limited to a specific region of the gastrointestinal tract. Regarding invasiveness, the tissue required for IHC is obtained from biopsies during standard gastroscopy or colonoscopy—procedures that are widely performed, technically mature, and associated with low risk. In contrast, obtaining tissue from the characteristic small intestinal ulcers often necessitates more complex procedures (e.g., balloon-assisted enteroscopy) or surgery, involving significantly greater technical difficulty and higher complication risks and cost. Furthermore, the economic benefit of IHC is substantial. In our setting, the approximate cost for SLCO2A1 immunohistochemistry is around 50 RMB per stain, whereas whole-exome or targeted genetic sequencing for CEAS diagnosis typically costs over 3000 RMB. Compared to whole-exome sequencing, which is currently relied upon for definitive diagnosis, IHC can typically be completed within 2–3 working days in a routine pathology laboratory, whereas genetic test results often require a turnaround time of several weeks. This two-order-of-magnitude cost difference, combined with the shorter turnaround time, makes IHC a highly accessible and practical initial test or screening tool, particularly in resource-conscious clinical environments. This broad applicability of IHC further supports its use as a feasible diagnostic tool for CEAS.

To date, numerous mutations spanning the entire gene have been reported, including splicing site, missense, nonsense, and frameshift mutations, with no single predominant hotspot variant. In our cohort, we identified 11 variants in 10 patients, which aligns with this known genetic heterogeneity. The most frequently reported mutation in the literature, the splicing site variant c.940 + 1G > A^2^, was also the most common in our study. Notably, we discovered seven novel variants not previously reported in CEAS patients, including c.1633A(2 > 1), c.581A > G, c.268_279del, and c.910_914del, contributing to the growing body of evidence on the genetic diversity of CEAS. Two patients in our cohort exhibited the same mutation type (c.940 + 1G > A), while the other mutations were unique to individual patients. This level of genetic diversity suggests that CEAS is not caused by a single mutation, but rather a range of genetic alterations in the SLCO2A1 gene, making it challenging to identify a single mutation that could serve as a diagnostic marker for all cases of the disease.

The clinical characteristics of the 10 CEAS patients in this cohort were largely consistent with previously reported cases [7], with common symptoms including abdominal pain (70%), melena (60%), ileus (50%), and hypoalbuminemia (100%). These symptoms, particularly abdominal pain and ileus, are frequently seen in chronic enteropathies, leading to misdiagnosis, as seen in our cohort, where 90% of patients were initially misdiagnosed with Crohn’s disease (CD). Consistent with previous reports demonstrating a male predominance in PHO [13,16], all three PHO cases in our cohort were male. This observation may suggest that SLCO2A1 loss-of-function mutations exert sex-hormone-related effects. Therefore, the presence of digital clubbing and PHO facial features in male patients should raise clinical suspicion for CEAS.

Our study has several limitations. First, the small sample size (*n* = 10), while reflective of the rarity of CEAS, limits the statistical power of our analysis. Second, the retrospective, single-center design may introduce selection bias and limits the completeness of uniformly collected data. Third is the lack of a formal control group (e.g., patients with Crohn’s disease, CMUSE, or healthy mucosa), which is essential to definitively establish the diagnostic specificity of SLCO2A1 IHC. Future multi-center, prospective studies with larger cohorts, including the necessary control cohorts, using standardized IHC protocols and blinded interpretation, are necessary to validate the diagnostic accuracy and establish robust clinical guidelines for implementing SLCO2A1 IHC.

## 5. Conclusions

Our findings support the potential utility of SLCO2A1 IHC as a promising, cost-effective, and accurate diagnostic tool for CEAS, with two complementary but distinct applications. First, in resource-limited settings, IHC on routinely available endoscopic biopsies (even from non-lesional sites like the gastric antrum or terminal ileum) could serve as a practical and cost-effective screening or presumptive diagnostic alternative. Second, in settings where genetic testing is available, IHC can act as a valuable supplemental and rapid adjunct. It could provide preliminary supportive evidence while awaiting genetic results and help in the interpretation of variants of uncertain significance found in SLCO2A1. IHC, when used in combination with clinical evaluation and genetic testing, could significantly enhance the diagnostic approach to CEAS, leading to earlier recognition and mitigating misdiagnosis.

## Figures and Tables

**Table 1:** Characteristics of CEAS patients.

Characteristics	N = 10
Gender, male, n (%)	4 (40%)
Age at symptom onset, median (IQR), yr	25 (17–36)
Age at diagnosis, median (IQR), yr	41 (39–44)
Interval from symptom onset to diagnosis, median (IQR), yr	15 (6–24)
Family history of CEAS, n (%)	0 (0%)
Parental consanguineous marriage	4 (40%)
History of bowel resection, n (%)	5 (50%)
Once	4 (40%)
Twice	1 (10%)
Interval from symptom onset to first bowel resection surgery median (IQR), yr	13 (1–26)
Symptoms and laboratory findings, n (%)	
Abdominal pain	7 (70%)
Diarrhea	1 (10%)
Ileus	5 (50%)
Melena	6 (60%)
Hematochezia	2 (20%)
Hypoalbuminemia	10 (100%)
Involved GI tract, n (%)	
Esophagus	0 (0%)
Stomach	0 (0%)
Duodenum	1 (10%)
Jejunum	1 (10%)
Ileum	10 (100%)
Colon	1 (10%)
PHO diagnosis	2
Male	2 (100%)
Female	0 (0%)
Clinical manifestation of PHO, n (%)	
Digital clubbing	2 (100%)
Pachydermia	2 (100%)
Periostosis	2 (100%)
Joint pain	2 (100%)
Laboratory data, median (IQR)	
Hemoglobin, g/L	70 (39.8, 100.5)
Albumin, g/L	28.02 (22.1, 33.0)
CRP, mg/L	10.24 (5.1, 13.5)
ESR, mm/hr	17.0 (5.0, 26.0)
Platelet, 10^9^/L	261 (141.5, 292.5)
Treatment history, n (%)	
Mesalazine	4 (40%)
Methylprednosolone	7 (70%)
Azathioprine	2 (20%)
Thalidomide	4 (40%)
Biologics	2 (20%)
Enteral nutrition	6 (60%)
Misdiagnosis	
CD	9 (90%)
CMUSE	4 (40%)
Undetermined	1 (10%)

**Table 2:** Identified SLCO2A1 gene mutations in CEAS patients.

Patients	Genomic Position chr3 (hg19)	Site	Mutation Type	Nucleotide Change	Mutation Site Type	Amino Acid Change	Other CEAS Reported	HGMD Reported
1	134029703	Intron 1	Homo	c.96 + 4A > C	SS	NA	No	Yes
2	133670050	Intron 6	Homo	c.861 + 2T > C	SS	NA	No	Yes
3	133657328 133672505	Exon 12 Intron 5	Compound Hetero	c.1633A [2 > 1] c.724 + 1G > A	FS SS	p.N545Tfs*15 NA	No No	No Yes
4	133673854 133692625	Exon 4 Exon 3	Compound Hetero	c.581A > G c.268_279del	MIS FS	p.Y194C p.Y90_S93del	No No	No No
5	133667736 133667763	Intron 7 Exon 7	Compound hetero	c.940 + 1G > A c.910_914del	SS FS	NA p.S304Rfs*8	Yes No	Yes No
6	133667736	Intron 7	Homo	c.940 + 1G > A	SS	NA	Yes	Yes
7	133935781	Exon 13	Homo	c.1807C > T	NON	p.R603*	Yes	Yes
8	133947445	Exon 9	Homo	c.1106G > A	MIS	p.G369D	Yes	Yes
9	133748547	Intron 1	Homo	c.96 + 4A > C	SS	NA	No	Yes
10	133667545	Intron 7	Homo	c.941-1G > A	SS	NA	Yes	Yes

Homo, Homozygous; Hetero, Heterozygous; MIS, Missense; NON, Nonsense; SS, Splicing Site; FS, Frame Shift; NA, Not Available; HGMD, Human Gene Mutation Database. *, Termination code.

**Table 3:** Symptoms and IHC staining results in CEAS patients.

Patients	Symptoms and Laboratory Findings	IHC Staining Results in Ulcer Region	IHC Staining Results in Other Gastrointestinal Regions
1	abdominal pain, ileus, diarrhea, hematochezia, hypoalbuminemia	absent	absent
2	abdominal pain, ileus, melena, hypoalbuminemia	absent	absent
3	hypoalbuminemia	absent	absent
4	abdominal pain, melena, hypoalbuminemia	absent	absent
5	melena, hypoalbuminemia	absent	absent
6	abdominal pain, ileus, melena, hypoalbuminemia	absent	absent
7	abdominal pain, melena, hypoalbuminemia	absent	absent
8	abdominal pain, ileus, hypoalbuminemia	reduced	reduced
9	abdominal pain, ileus, melena, hypoalbuminemia	absent	absent
10	hematochezia, hypoalbuminemia	absent	absent

## Data Availability

The original contributions presented in this study are included in the article. Further inquiries can be directed to the corresponding author.

## References

[1] Umeno J., Esaki M., Hirano A., Fuyuno Y., Ohmiya N., Yasukawa S., Hirai F., Kochi S., Kurahara K., Yanai S. (2018). Clinical features of chronic enteropathy associated with SLCO2A1 gene: A new entity clinically distinct from Crohn’s disease. J. Gastroenterol..

[2] Umeno J., Hisamatsu T., Esaki M., Hirano A., Kubokura N., Asano K., Kochi S., Yanai S., Fuyuno Y., Shimamura K. (2015). A Hereditary Enteropathy Caused by Mutations in the SLCO2A1 Gene, Encoding a Prostaglandin Transporter. PLoS Genet..

[3] Nakanishi T., Tamai I. (2017). Roles of Organic Anion Transporting Polypeptide 2A1 (OATP2A1/SLCO2A1) in Regulating the Pathophysiological Actions of Prostaglandins. AAPS J..

[4] Nakanishi T., Nakamura Y., Umeno J. (2021). Recent advances in studies of SLCO2A1 as a key regulator of the delivery of prostaglandins to their sites of action. Pharmacol. Ther..

[5] Nakata R., Nakamura Y., Hosomi S., Okuda H., Nishida Y., Sugita N., Itani S., Nadatani Y., Otani K., Tanaka F. (2020). Slco2a1 deficiency exacerbates experimental colitis via inflammasome activation in macrophages: A possible mechanism of chronic enteropathy associated with SLCO2A1 gene. Sci. Rep..

[6] Hosoe N., Ohmiya N., Hirai F., Umeno J., Esaki M., Yamagami H., Onodera K., Bamba S., Imaeda H., Yanai S. (2017). Chronic Enteropathy Associated With SLCO2A1 Gene [CEAS]-Characterisation of an Enteric Disorder to be Considered in the Differential Diagnosis of Crohn’s Disease. J. Crohn’s Colitis.

[7] Hong H.S., Baek J., Park J.C., Lee H.S., Park D., Yoon A.R., Park S.J., Hong S.N., Koh S.J., Lee C.K. (2022). Clinical and Genetic Characteristics of Korean Patients Diagnosed with Chronic Enteropathy Associated with SLCO2A1 Gene: A KASID Multicenter Study. Gut Liver.

[8] Shang Q., Dai Y., Huang J., Liu W., Zhou W., Liu Y., Yang H., Wang Q., Li Y. (2024). Clinical and genetic characteristics of Chinese patients diagnosed with chronic enteropathy associated with SLCO2A1 gene. Orphanet J. Rare Dis..

[9] Jeong B., Park S.H., Ye B.D., Kim J., Yang S.K. (2023). A Novel Chronic Enteropathy Associated with SLCO2A1 Gene Mutation: Enterography Findings in a Multicenter Korean Registry. Korean J. Radiol..

[10] Perlemuter G., Guillevin L., Legman P., Weiss L., Couturier D., Chaussade S. (2001). Cryptogenetic multifocal ulcerous stenosing enteritis: An atypical type of vasculitis or a disease mimicking vasculitis. Gut.

[11] Helmy M., Awad M., Mosa K.A. (2016). Limited resources of genome sequencing in developing countries: Challenges and solutions. Appl. Transl. Genom..

[12] Xin L., Liao Z., Jiang Y.P., Li Z.S. (2011). Indications, detectability, positive findings, total enteroscopy, and complications of diagnostic double-balloon endoscopy: A systematic review of data over the first decade of use. Gastrointest. Endosc..

[13] Lu Q., Xu Y., Zhang Z., Li S., Zhang Z. (2023). Primary hypertrophic osteoarthropathy: Genetics, clinical features and management. Front. Endocrinol..

[14] Matsuno Y., Umeno J., Esaki M., Hirakawa Y., Fuyuno Y., Okamoto Y., Hirano A., Yasukawa S., Hirai F., Matsui T. (2019). Measurement of prostaglandin metabolites is useful in diagnosis of small bowel ulcerations. World J. Gastroenterol..

[15] Yamaguchi S., Yanai S., Nakamura S., Kawasaki K., Eizuka M., Uesugi N., Sugai T., Umeno J., Esaki M., Matsumoto T. (2018). Immunohistochemical differentiation between chronic enteropathy associated with SLCO2A1 gene and other inflammatory bowel diseases. Intest. Res..

[16] Yuan L., Chen X., Liu Z., Wu D., Lu J., Bao G., Zhang S., Wang L., Wu Y. (2018). Novel SLCO2A1 mutations cause gender-differentiated pachydermoperiostosis. Endocr. Connect..

